# Gender Differences in Insomnia and Role of Work Characteristics and Family Responsibilities Among Healthcare Workers in Taiwanese Tertiary Hospitals

**DOI:** 10.3389/fpsyt.2022.831075

**Published:** 2022-04-29

**Authors:** Meng-Ting Tsou

**Affiliations:** ^1^Department of Family Medicine, MacKay Memorial Hospital, Taipei City, Taiwan; ^2^Department of Occupation Medicine, MacKay Memorial Hospital, Taipei City, Taiwan; ^3^Department of Nursing, MacKay Junior College of Medicine, Nursing, and Management, New Taipei City, Taiwan

**Keywords:** athens insomnia scale, family responsibility, gender difference, insomnia, work characteristics

## Abstract

**Background:**

Insomnia is common among healthcare workers (HCWs), especially those working in tertiary hospitals. This study aimed to clarify whether gender differences in insomnia could be explained by gender differences in work characteristics and family responsibilities among HCWs in tertiary hospitals in Taiwan.

**Methods:**

This cross-sectional study was conducted in 22 departments of two tertiary hospitals in Northern Taiwan from December 2018 to March 2019. All data were obtained by a self-administered questionnaire given when participants underwent annual health check-ups. Insomnia was evaluated using the Chinese Athens Insomnia Scale. Work characteristics and family responsibilities were as follows: department, working hours, shift work, visual display terminals used at work, demand-control-support model, burnout level, breadwinner status, living conditions, and caregiver status. Data of 2,811 participants (317 men, 11.3%; 2,494 women, 88.7%) were analyzed (response rate: men, 85%; women, 88%). Logistic regression analysis examined howwork characteristics and family responsibilities explained gender differences in insomnia.

**Results:**

The prevalence of insomnia in women (61.7%) was significantly higher than that in men (52.7%), and gender differences strengthened after adjusting for work characteristics and family responsibilities [odds ratio: 1.45 (1.11–1.90) and 1.62 (1.18–2.22), *p* < 0.01]. Stratified analyses revealed that significant gender differences were found among HCWs with comparatively unfavorable work and family conditions. Furthermore, women had a higher association of insomnia owing to these factors.

**Conclusion:**

These results suggest that gender differences in insomnia among HCWs are mainly explained by gender differences in work characteristics and family responsibilities.

## Introduction

Sleep problems, especially insomnia, are a common public health concern in many countries (1). The prevalence in the general population ranged from 2.3 to 25.5% (1). Several studies have reported a higher prevalence of sleep problems among healthcare workers, including 21–65.5% in China (2, 3), 30.7% in Japan (4), 43% in Iran (5), and 73.4% in the United States (6). In addition, up to 40% in India and 48.5% in China of HCWs in tertiary hospitals who had long working hours and shift works were likely to cause insomnia (7, 8).

Paying attention to sleep problems is crucial because it affects the health and safety of healthcare workers (HCWs). Several studies have confirmed that sleep problems significantly affect the risk of cardiovascular disease, immune disorders, cancer, and anxiety (9–11) and are also associated with an increased risk of work-related injuries, such as needle stick injuries (12, 13). In addition, lack of sleep caused by long working hours and shift works affects patient safety, including increased surgical complications, adverse drug events, and misdiagnoses (14–16).

A meta-analysis of Zhang et al. and Zeng et al. reported that women had a significantly higher prevalence of insomnia than men (17, 18). Insomnia is 1.3–2.0 times more common in women than in men (19). There are three possible explanations for this gender difference in the prevalence of insomnia: biological differences, prior psychiatric illnesses, and sociological differences (19, 20). First, biological explanations suggest that innate physiological differences between men and women cause insomnia. Studies have shown that sex steroids, such as progesterone, estrogen, and testosterone can affect the difference in sleep patterns between men and women (21). Second, mental illnesses such as anxiety and depression can also cause insomnia (22) and are more common in women than in men (23, 24). Third, the sociological approach to insomnia focuses on gender inequality in work characteristics and family responsibilities (25). Many studies have found that gender differences impact health (25–27). Some studies have examined social factors, including work characteristics and family responsibilities, which could explain differences in gender insomnia; however, the results are inconsistent (19, 28). Therefore, in contrast to biological and psychiatric differences, the study of sociological differences has not been completely investigated.

This study specifically targeted HCWs in tertiary hospitals. The study aimed to understand the prevalence of insomnia among the different genders and clarify whether work characteristics and family responsibilities cause gender differences in insomnia rates s. We considered a broad range of factors, including occupational stress dimensions (based on Karasek's demand-control-support model) (29), working hours, shift work responsibilities, visual display terminals (VDTs) using at work, breadwinner status, childcare or disabled family care responsibilities, and caregiver status.

## Methods

### Participants and Data Collection

This cross-sectional study was conducted at the Health Evaluation Center in MacKay Memorial Hospital, a 2,000-bed tertiary teaching center in Taipei/New Taipei, Taiwan branch. This study was a voluntary response sample. Questionnaires were collected between December 2018 and March 2019 during the employees' annual physical check-ups. The contents of the self-administered questionnaire were explained to the participants before administration. If more than one-fourth of the questionnaire was not completed, the questionnaire was excluded.

**Figure 1:**
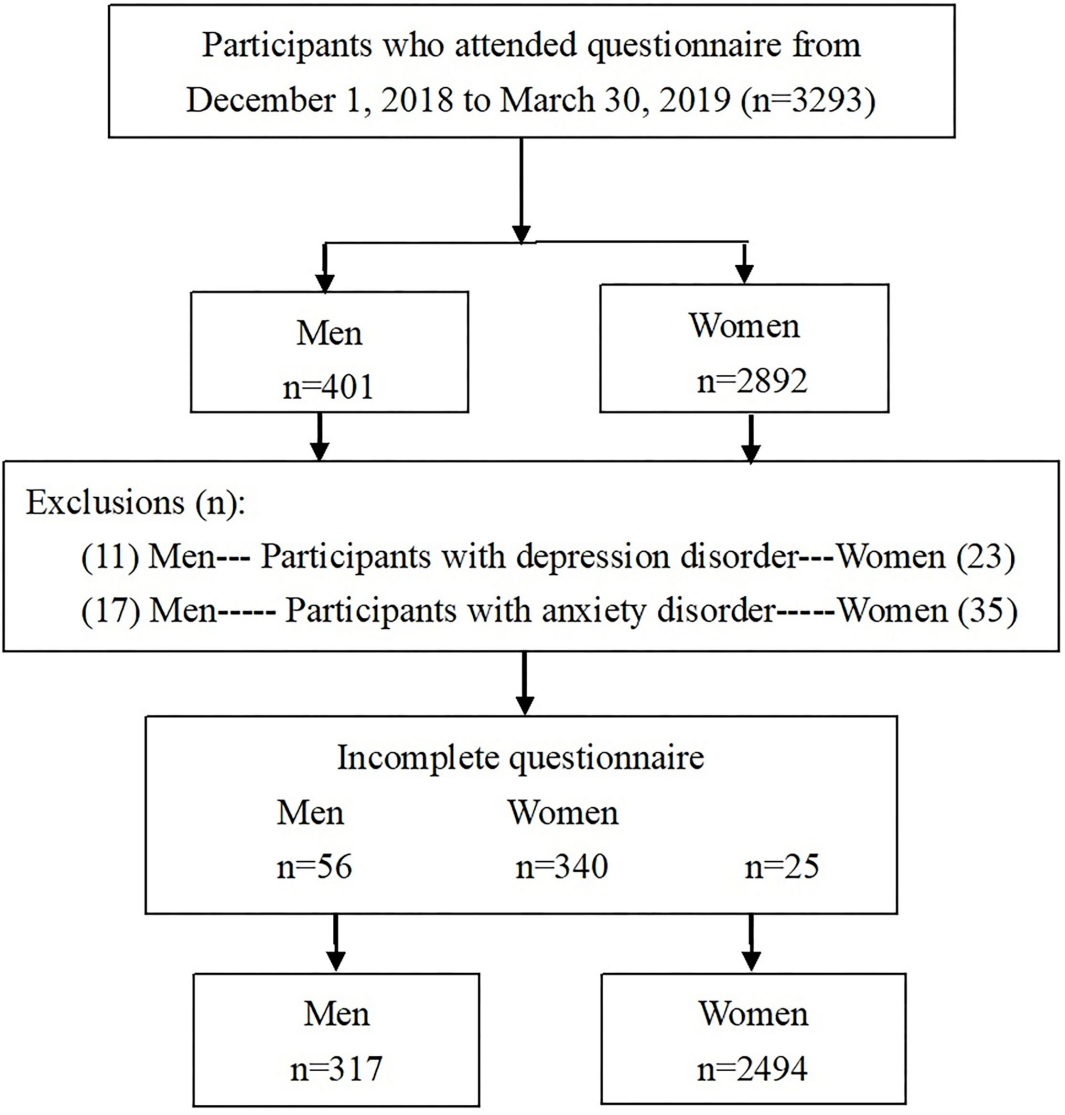
Selection process of participants.

373 men and 2,834 women were examined after excluding individuals (Figure 1). Overall, 317 were men and 2,494 were women who completed the questionnaire after excluding anxiety or depression disorders, and the actual response rates were 84.9% and 88.0%, respectively. A sample size of 2,533 achieved 90% power using a two-tailed test (odd ratio [OR] = 1.59, probability of null hypothesis = 0.15, alpha error = 0.05, power = 0.9, and R^2^ for other confounding factors = 0.5) (30).

Information was collected using structured questionnaires designed according to the instructions of the Institute of Labor, Occupational Safety, Ministry of Labor (Survey of Perceptions of Safety and Health in the Work Environment in 2016 Taiwan; ILOSH105–M309) (31). Analysis of the internal consistency reliability of this questionnaire showed Cronbach's α to be between 0.82 and 0.93, indicating good reliability. In addition, the following data were collected: socioeconomic status, lifestyle, work characteristics, job burnout syndrome status, and family responsibilities. Cronbach's α was between 0.80 and 0.92 in our study.

### Assessment of Insomnia

We assessed insomnia using the Chinese Version of the Athens Insomnia Scale (CAIS). The CAIS is based on the International Classification of Disease, 10th Revision (ICD-10) criteria and has been validated (32). The scale is a self-administered inventory consisting of eight items (CAIS-8). The first five items assess difficulties in sleep induction, awakening during the night, final awakening earlier than desired, total sleep duration, and overall sleep quality. The remaining three items pertain to next-day consequences of insomnia, including the sense of well-being, functioning, and sleepiness during the day. Each item of the CAIS can be rated from 0 (no problem at all) to 3 (very serious problem), and the total score ranges from 0 to 24. The Cronbach's α of the internal consistency reached 0.82–0.84 in original study (33), and 0.80–0.83 in our study. The correlation coefficients of test-retest reliability were 0.84–0.86 (33). The suggested cutoff points for insomnia among the ethnic Chinese population were 8 for the CAIS-8 (AUC = 0.89, *p* < 0.001), and participants were divided into two categories: ≥ 8 (presence of insomnia); and < 8 (absence of insomnia) (33). Respondents were asked to calculate their score if they experienced sleep difficulties at least three times a week during the previous month. Insomnia may be classified according to the duration of difficulty sleeping into 3 groups: short-term insomnia disorder (< 3 months), chronic insomnia disorder (sleep disturbances that occur at least three times per week for > 3 months), and other insomnia disorder (32). The definition of insomnia in our study belonged to “short-term insomnia”.

### Burnout Assessment

Burnout domains were evaluated using the Chinese version of the Maslach Burnout Inventory–Health Services Survey test (MBI-HSS) as per Lu et al. (Cronbach's α, 0.84) (34). Three domains of the MBI-HSS were evaluated: emotional exhaustion (EE); depersonalization (DP); and personal accomplishment (PA). The MBI-HSS includes 22 questions in total, and the frequency of occurrence is scored using a 7-point Likert scale from 0 (never felt) to 6 (felt every day). EE scores ≥ 27, DP scores ≥ 13, and PA scores ≤ 31 were categorized as high job burnout, in which EE and DP were directly related to burnout, whereas PA showed a negative association (34). The internal consistency coefficients (Cronbach's α) of the EE, DP, and PA dimensions of original Chinese version of MBI-HSS were 0.77, 0.83, and 0.82, respectively (34). The internal consistency coefficients (Cronbach's α) of the EE, DP, and PA dimensions of the current study were 0.79, 0.81, and 0.80, respectively.

### Assessments of Work Characteristics

Fundamental work-related issues included the department and seniority. Workforces in all departments, such as doctors, nurses, technicians, administrative staff, pharmacists, radiologists, and nutritionists, were also included. Since nurses and doctors have similar work characteristics, including shift works and excessive working hours (35), they were categorized as the doctor/nurse group, and the remaining staff were categorized as the non-doctor/nurse group for the analysis.

Information about working hours and shift works was derived from the work status provided by the participants during the month before the survey. Working hours (h/week) were grouped into <50 h/week and 50 h/week or more. Shift work was classified as either yes or no. The duration of daily VDTs used at work was categorized into two categories: <6 h/day; and 6 h/day or more (36). Occupational stress was assessed using the demand-control-support model (DCS) (37), in the Chinese version of the Job Content Questionnaire based on the Karasek's job strain model (29, 38).

The Chinese version of the DCS model consists of five items for the demand scale, nine items for the job control scale, and eight items for occupation support (38). Each item has four agreement-based response categories ranging from strongly disagree (one point) to strongly agree (four points) on a Likert scale. Scores for job demand, job control, and occupational support were obtained using weighted calculations. Job strain was defined as the ratio of job demand to job control. The job strain scores were divided into three, and we defined the upper tertile as the high job strain group (39). The Cronbach's alpha coefficient was approximately 0.65 for the three subscales among the different genders (38), about 0.62 in our study.

### Measures for Family Characteristics

Breadwinner status, living conditions, childcare or disabled family care, and caregiver status were chosen to measure family responsibilities. Breadwinner status was determined by a simple yes or no question. Living conditions were divided into two groups: individuals living alone; and those living with family or friends. Regarding childcare or disabled family care, participants were asked to answer whether they had preschool children or a disabled family member living with them (yes or no). Preschool children were classified as children aged <6 years. Disabled family members were defined as people who were totally or partially bedridden. Finally, regarding caregiver status, participants were asked to answer whether they were the main person caring for and supporting their families (yes or no) (31).

### Other Putative Confounding Factors

Educational background was categorized into high school education or less and more than high school education (college or graduate school). Anthropometric measurements (height and body weight) were recorded using a standardized protocol. Height was measured with a standard stadiometer with an approximation value of 0.01 cm. Weight was measured using a set of calibrated electronic scales. The participants wore light clothes weighing approximately 0.01 kg. These procedures were performed before 10:00 AM. Height and weight measurements were performed and recorded by trained nurses working at the Health Evaluation Center and were blinded to patients' information. Body mass index (BMI) was calculated as weight (kg)/height (m^2^) and was divided into four groups: < 18.5, 18.5–24, 24–27, and ≥ 27 (40).

Information concerning personal habits, including smoking status (yes/no: current or past/never) and alcohol consumption (times/week), were divided into three categories: < 2 times/week, 3–4 times/week, and ≥ 5 times/week. Exercise frequency (times/week) was divided into four categories: never, 1 time/week, 2–4 times/week, ≥ 5 times/week, and ≥ 30 min/set (31).

### Ethics Approval

The study protocol was reviewed and approved by the Research Ethics Committee on Human Beings at the MacKay Memorial Hospital (Research Project Number 18MMHISO150).

All participants provided written informed consent. Confidentiality in data collection was preserved. This study was conducted in accordance with the guidelines of the Declaration of Helsinki. To ensure data confidentiality, patient identification information was replaced with a sheet number to ensure data confidenytiality.

### Statistical Analysis

First, chi-square tests were used to evaluate whether gender differences were present in age, work characteristics, family responsibilities, and lifestyle factors. Next, the values in the two groups were assessed according to the CAIS and compared using the chi-squared test performed separately for men and women. Then, a logistic regression analysis was performed to analyze the influence of work characteristics and family responsibilities on gender differences in insomnia. Explanatory variables were added sequentially to a logistic regression model that included gender.The changes in the OR in gender were used to evaluate the impact of subsequently introduced variables on gender differences in insomnia. We introduced the following variables into each logistic regression model: demographic and lifestyle factors (age, education, smoking, drinking, exercise, and BMI; Model 1); work characteristics (department, seniority, working hours, shift work, duration of VDT used at work, job strain, occupational support, and burnout) and Model 1(Model 2); family responsibilities (breadwinner, living condition, childcare or disabled family care, and caregiving) and Model 1(Model 3); and work characteristics, family responsibilities, and Model1 (Model 4). Finally, to examine whether work characteristics and family responsibilities modified gender differences in insomnia, multiple logistic analysis and interaction analysis by variables significantly associated with insomnia in Model 4 were performed after adjusting for factors. Statistical analysis was performed using the Statistical Package for the Social Sciences Statistics version 22 (IBM Corp., Armonk, NY, USA) for Windows, and *p* < 0.05 was considered bilateral.

## Results

### Characteristics of the Participants

The characteristics of the study participants are presented in Table 1. There are more women in the doctor/ nurse group than men (69.25% vs. 43.85%, p < 0.001). More women have insomnia than men (61.23% vs. 52.68%, *p* = 0.003). Women who reported insomnia were on average younger than men those who reported insomnia (38.24 vs. 39.90 years, *p* = 0.020). In terms of lifestyle, including 13.88% for men who smoked (women: 2.37%, *p* < 0.001), 2.84% for men who drank (women: 0.72%, *p* = 0.001), 66.46% for men exercised weekly (women: 50.84%, *p* < 0.001), and 57.82% for men who were overweight and obese (women: 34.19%, *p* < 0.001), the proportion for men was higher than that of women.

**Table 1:** Characteristics of men and women participants.

**Variables**	**Men** **(*n* = 317)**	**Women** **(*n* = 2494)**	***p*-value**
Age, years	39.90 ± 11.33	38.24 ± 11.95	0.020
Department, *n* (%)			<0.001
Non-doctor/nurse	178 (56.15)	767 (30.75)	
Doctor/nurse	139 (43.85)	1727 (69.25)	
Insomnia, *n* (%)			0.003
Non-insomniacs	150 (47.32)	967 (38.77)	
Insomniacs	167 (52.68)	1527 (61.23)	
Age, years, *n* (%)			0.687
<39	176 (55.52)	1375 (55.13)	
40–44	37 (11.67)	346 (13.87)	
45–49	30 (9.46)	243 (9.74)	
50–54	32 (10.09)	261 (10.47)	
55–59	26 (8.20)	180 (7.22)	
≧60 s	16 (5.05)	89 (3.57)	
Education level, *n* (%)			0.838
High school education or less	43 (13.56)	328 (13.15)	
More than high school education (college+ graduate school)	274 (86.44)	2166 (86.85)	
Alcohol consumption, times/week, *n* (%)			0.001
<2	308 (97.16)	2476 (99.28)	
3–4	8 (2.52)	12 (0.48)	
≧5	1 (0.32)	6 (0.24)	
Smoking, *n* (%)	44 (13.88)	59 (2.37)	<0.001
Exercise frequency, times/week, *n* (%)			<0.001
Never	106 (33.44)	1226 (49.16)	
1	89 (28.08)	668 (26.78)	
2–4	103 (32.49)	534 (21.41)	
≧5	19 (5.99)	66 (2.65)	
Body mass index, kg/m^2^, *n* (%)			<0.001
<18.5	5 (1.60)	224 (9.09)	
18.5–24	127 (40.58)	1397 (56.72)	
24–27	92 (29.39)	450 (18.27)	
≧27	89 (28.43)	392 (15.92)	
Seniority, years, *n* (%)			0.001
<4	139 (43.99)	860 (34.48)	
≧4	177 (56.01)	1634 (65.52)	
Working hours/week, *n* (%)			<0.001
<50	259 (81.96)	2330 (93.42)	
≧50	57 (18.04)	164 (6.58)	
Shift work, *n* (%)			0.115
No	159 (50.16)	1134 (45.47)	
Yes	158 (49.84)	1360 (54.53)	
Duration of VDT work, hour/day, *n* (%)			<0.001
<6	238 (75.08)	1281 (51.36)	
≧6	79 (24.92)	1213 (48.64)	
Demand-Control-Model (DCM)			
Job demand	64.40 ± 17.99	71.46 ± 17.08	<0.001
Job control	55.56 ± 12.99	55.67 ± 11.11	0.864
Job strain (job demand/job control)	1.26 ± 0.70	1.34 ± 0.51	<0.001
Job strain (job demand/job control), *n* (%)			0.010
Low	51 (16.09)	155 (6.22)	
Middle	253 (79.81)	2235 (89.69)	
High	13 (4.10)	102 (4.09)	
Occupation support	61.11 ± 12.57	62.25 ± 10.41	0.074
Occupation support, *n* (%)			0.008
Low	15 (4.75)	54 (2.17)	
Middle	240 (75.95)	2016 (81.19)	
High	61 (19.30)	413 (16.63)	
MBI			
Emotional Exhaustion	21.47 ± 10.70	25.72 ± 10.79	<0.001
Depersonalization	9.69 ± 5.94	10.68 ± 5.84	0.005
Personal accomplishment	26.67 ± 6.24	26.72 ± 5.34	0.880
Burnout, *n* (%)			0.742
No	295 (93.06)	2333 (93.54)	
Yes	22 (6.94)	161 (6.46)	
Breadwinner, *n* (%)			<0.001
No	60 (28.85)	1113 (52.48)	
Yes	148 (71.15)	1008 (47.52)	
Living condition, *n* (%)			0.661
Single	19 (9.13)	214 (10.09)	
With family or friend	189 (90.87)	1907 (89.91)	
Childcare or disability family, *n* (%)			0.701
No	162 (77.88)	1627 (76.71)	
Yes	46 (22.12)	494 (23.29)	
Caregiver, *n* (%)			0.427
No	182 (87.50)	1813 (85.48)	
Yes	26 (12.50%)	308 (14.52)	

*MBI-HSS, Maslach Burnout Inventory–Health Services Survey test; VDT, visual display terminal; SD, standard deviation*.

*Clinical characteristics are expressed as mean ± SD for continuous variables and n (%) for categorical variables. P-value was derived from independent two-sample t-test for continuous variables and categorical variables were expressed as percentages and compared with the x^2^ test*.

65.52 % for women who had more than 4 years of seniority (men: 56.01%, *p* = 0.001), 48.64% for women who spent more than 6 h of VDT used at work (men: 24.92%, *p* < 0.001), job demands (women vs. men: 93.8 vs. 87.2, *p* < 0.001), and job strain (women vs. men: 1.33 vs. 1.24, *p* < 0.001), the proportion for women was higher than that of men. In addition, in two domains (EE and DP), the scores were significantly higher in women than in men. However, there was no statistically significant difference in the proportion of burnout between women and men (6.46% vs. 6.94%, p = 0.742).

The proportion of men working more than 50 h/week was relatively high compared to women (18.04% vs. 6.58%, *p* < 0.001). Men were polarized in occupational support, in which the proportions of low and high support were both higher. Women had moderate occupational support, and men were usually the primary source of income in the family, accounting for 71.15%. There was no statistically significant difference between men and women in childcare or disabled family care and caregiver status.

To compare to women without insomnia, Table 2 shows that the higher proportion of women with insomnia was in the two age groups (< 39 years: 58.48% vs. 49.84% and 40–44 years: 14.54% vs. 12.82%, *p* < 0.001); and in the doctor/nurse group (71.25% vs. 66.08%, p < 0.001). The age of women with insomnia was lower than that of women without insomnia (37.40 vs. 39.58 years, p < 0.001). In addition, a higher education level (more than college) was found among women with insomnia (89.0% vs. 83.45%, *p* < 0.001). However, the weekly exercise level was lower in women with insomnia than those without insomnia (53.24% vs. 42.71%, *p* < 0.001). There were no significant differences in men with and without insomnia for the department, education level, lifestyle, and seniority.

**Table 2:** Comparison of insomnia with non-insomnia by gender.

**Variables**	**Men**			**Women**		

	**Non-insomnia**	**Insomnia**	***p*** **value**	**Non-insomnia**	**Insomnia**	***p*** **-value**
	**(*****n*** **=** **99)**	**(*****n*** **=** **109)**		**(*****n*** **=** **811)**	**(*****n*** **=** **1310)**	
Department, *n* (%)			0.392			0.006
Non-doctor/nurse	88 (58.67)	90 (53.89)		328 (33.92)	439 (28.75)	
Doctor/nurse	62 (41.33)	77 (46.11)		639 (66.08)	1088 (71.25)	
Age, years	40.55 ± 12.00	39.31 ± 10.70	0.335	39.58 ± 12.48	37.40 ± 11.52	<0.001
Age, years, *n* (%)			0.689			<0.001
<39	80 (53.33)	96 (57.49)		482 (49.84)	893 (58.48)	
40–44	17 (11.33)	20 (11.98)		124 (12.82)	222 (14.54)	
45–49	13 (8.67)	17 (10.18)		110 (11.38)	133 (8.71)	
50–54	15 (10.00)	17 (10.18)		120 (12.41)	141 (9.23)	
55–59	15 (10.00)	11 (6.59)		86 (8.89)	94 (6.16)	
≧60	10 (6.67)	6 (3.59)		45 (4.65)	44 (2.88)	
Education level, *n* (%)			0.383			<0.001
High school education or less	23 (15.33)	20 (11.98)		160 (16.55)	168 (11.00)	
More than high school education (college + graduate school)	127 (84.67)	147 (88.02)		807 (83.45)	1359 (89.00)	
Alcohol consumption, times/week, *n* (%)			0.726			0.429
1–2	147 (98.00)	161 (96.41)		963 (99.59)	1513 (99.08)	
3–4	3 (2.00)	5 (2.99)		3 (0.31)	9 (0.59)	
≧5	0 (0.00)	1 (0.60)		1 (0.10)	5 (0.33)	
Smoking, *n* (%)	20 (13.33)	24 (14.37)	0.790	17 (1.76)	42 (2.75)	0.112
Exercise frequency (times/week), *n* (%)			0.211			<0.001
never	48 (32.00)	58 (34.73)		413 (42.71)	813 (53.24)	
1	36 (24.00)	53 (31.74)		273 (28.23)	395 (25.87)	
2–4	57 (38.00)	46 (27.54)		241 (24.92)	293 (19.19)	
≧5	9 (6.00)	10 (5.99)		40 (4.14)	26 (1.70)	
Body mass index, kg/m^2^, *n* (%)			0.715			0.066
<18.5	3 (2.03)	2 (1.21)		78 (8.08)	149 (9.74)	
18.5–24	64 (43.24)	63 (38.18)		579 (59.92)	835 (54.70)	
24–27	40 (27.03)	53 (31.52)		171 (17.63)	285 (18.68)	
≧27	43 (27.70)	49 (29.09)		139 (14.38)	255 (16.89)	
Seniority, years, *n* (%)			0.311			0.152
<4	70 (46.98)	69 (41.32)		350 (36.19)	510 (33.40)	
≧4	80 (53.02)	98 (58.68)		617 (63.81)	1017 (66.60)	
Working hours/week, *n* (%)			<0.001			<0.001
<50	135 (90.00)	124 (74.70)		931 (96.28)	1399 (91.62)	
≧50	15 (10.00)	43 (25.30)		36 (3.72)	128 (8.38)	
Shift work, *n* (%)			0.284			<0.001
No	80 (53.33)	79 (47.31)		493 (50.98)	641 (41.98)	
Yes	70 (46.67)	88 (52.69)		474 (49.02)	886 (58.02)	
Duration of VDT work, hours/day, *n* (%)			0.254			<0.001
<6	117 (78.00)	121 (72.46)		551 (56.98)	730 (47.81)	
≧6	33 (22.00)	46 (27.54)		416 (43.02)	797 (52.19)	
Demand-Control-Model (DCM), *n* (%)						
Job demand	58.98 ± 16.51	69.26 ± 17.92	<0.001	66.92 ± 16.43	74.34 ± 16.87	<0.001
Job control	57.47 ± 12.11	53.73 ± 13.72	0.036	56.80 ± 10.82	52.59 ± 11.29	0.034
Job strain (job demand/job control)	1.12 ± 0.62	1.39 ± 0.75	<0.001	1.25 ± 0.49	1.40 ± 0.51	<0.001
Job strain (job demand/job control), *n* (%)			0.014			<0.001
Low	32 (21.33)	19 (11.38)		88 (9.12)	67 (4.39)	
Middle	116 (77.34)	137 (82.03)	847 (87.56)	1390 (91.03)	
High	2 (1.33)	11 (6.59)		32 (3.32)	70 (4.58)	
Occupation support	63.30 ± 11.57	60.04 ± 13.35	<0.001	63.22 ± 10.33	61.63 ± 10.42	<0.001
Occupation support, *n* (%)			0.516			0.111
Low	6 (4.03)	11 (6.59)		16 (1.66)	38 (2.50)	
Middle	116 (77.18)	126 (75.45)		791 (81.81)	1274 (83.44)	
High	28 (18.79)	30 (17.96)		160 (16.53)	215 (14.06)	
MBI, *n* (%)						
Emotional Exhaustion	17.28 ± 9.22	25.24 ± 10.56	<0.001	21.29 ± 9.36	28.52 ± 10.71	<0.001
Depersonalization	7.90 ± 5.07	11.31 ± 6.20	<0.001	9.00 ± 5.19	11.75 ± 5.97	<0.001
Personal accomplishment	27.11 ± 5.81	26.27 ± 6.58	0.236	26.77 ± 5.22	26.68 ± 5.42	0.689
Burnout, *n* (%)			<0.001			0.001
No	148 (98.67)	147 (88.02)		925 (95.66)	1408 (92.21)	
Yes	2 (1.33)	20 (11.98)		42 (4.34)	119 (7.79)	
Breadwinner, *n* (%)			0.454			0.005
No	47 (31.31)	44 (26.61)		545 (56.35)	765 (50.08)	
Yes	103 (68.69)	123 (73.39)		422 (43.65)	762 (49.92)	
Living condition, *n* (%)			0.154			0.536
Single	18 (12.12)	11 (6.42)		103 (10.60)	149 (9.77)	
With family or friend	132 (87.88)	156 (93.58)		864 (89.40)	1378 (90.23)	
Childcare or disability family, *n* (%)			0.021			<0.001
No	127 (84.85)	120 (71.56)		792 (81.87)	1122 (73.51)	
Yes	23 (15.15)	47 (28.44)		175 (18.13)	405 (26.49)	
Caregiver, *n* (%)			0.007			<0.001
No	141 (93.94)	136 (81.65)		874 (90.38)	1259 (82.44)	
Yes	9 (6.06)	31 (18.35)		93 (9.62)	268 (17.56)	

*MBI-HSS, Maslach Burnout Inventory–Health Services Survey test; VDT, visual display terminal; SD, standard deviation*.

*Clinical characteristics are expressed as mean ± SD for continuous variables and n (%) for categorical variables. P-value was derived from independent two-sample t-test for continuous variables and categorical variables were expressed as percentages and compared with the x^2^ test*.

The proportion and scores of the insomnia groups (both gender) were higher than those of the non-insomnia groups for many work characteristics, including working hours ≥ 50 h/week (men: 25.30% vs. 10.0%; women: 8.38% vs. 3.72%, *p* < 0.001), job demands, job control, and job strain. However, the occupational support score was lower in the insomnia group than in the non-insomnia group. Finally, the burnout rate (especially in two domains: EE; and DP scores) was higher in the insomnia group than in the non-insomnia group (Table 2).

In terms of childcare or disabled family care (men: 28.44% vs. 15.15%, *p* = 0.021; women: 26.49% vs. 18.13%, *p* < 0.001) and caregiver responsibilities (men: 18.35% bs. 6.06%, *p* = 0.007; women: 17.56% vs. 9.62%, *p* < 0.001), the proportion of men and women with insomnia was higher than that of those without insomnia. The proportion of breadwinners in the women with insomnia group was higher than that of those without insomnia group (49.94% vs. 43.65%. *p* = 0.005). Living conditions did not affect the presence or absence of insomnia (Table 2).

### OR of Gender Differences in Insomnia Before and After Adjusting for Work Characteristics and Family Responsibilities

Table 3 shows that the OR between women and men persisted after adjusting for work characteristics (Model 2) (Model 1 vs. Model 2: OR = 1.47 vs. 1.45, *p* = 0.003 vs. 0.003). The OR after adjusting for family responsibilities (Model 3) was significantly different (Model 3, OR = 1.62, *p* = 0.003). After adjusting for work characteristics and family responsibilities (Model 4), the OR was 1.48 (*p* = 0.019). Work characteristics and family responsibilities significantly associated with insomnia in Model 4 were seniorities (OR = 1.33, 95% CI: 1.05–1.79, *p* = 0.021), weekly working hours (OR = 2.27, 95% CI: 1.45–3.54, *p* < 0.001), shift work (OR = 1.26, 95% CI: 1.05–1.52, p = 0.029), VDT used at work (OR = 1.37, 95% CI: 1.13–1.65, *p* = 0.001), job strain (middle: OR = 1.65, 95% CI: 1.16–2.34, *p* = 0.004), burnout (OR = 1.87, 95% CI: 1.23–2.84, *p* = 0.004), breadwinner status (OR = 1.32, 95% CI: 1.09–1.59, *p* = 0.004), and caregiver status (OR = 1.46, 95% CI: 1.01–2.15, *p* = 0.041).

**Table 3:** Gender differences in insomnia before and after adjustment work and family characteristics.

**Variables**	**Model 1**		**Model 2**		**Model 3**		**Model 4**	
	**OR 95% CI**	***p*-value**	**OR 95% CI**	***p*-value**	**OR 95% CI**	***p*-value**	**OR 95% CI**	***p*-value**
Gender								
Men	Referent		Referent		Referent		Referent	
Women	1.47 (1.14–1.90)	0.003	1.45 (1.11–1.90)	0.013	1.62 (1.18–2.22)	0.003	1.48 (1.06–2.05)	0.019
Department								
Non-doctor/nurse				Referent			Referent	
Doctor/nurse			0.84 (0.68–1.02)	0.081			0.86 (0.69–1.07)	0.174
Seniority								
<4 years			Referent				Referent	
≧4 years			1.38 (1.12–1.71)	0.003			1.33 (1.05–1.69)	0.021
Working hours/week								
<50 h/week			Referent				Referent	
≧ 50 h/week			2.20 (1.56–3.10)	<0.001			2.27 (1.45–3.54)	<0.001
Shift work								
No			Referent				Referent	
Yes			1.25 (1.05–1.49)	0.013			1.26 (1.05–1.52)	0.023
Duration of VDT work								
<6 h/day			Referent				Referent	
≧ 6 h/day			1.34 (1.13–1.60)	0.001			1.37 (1.13–1.65)	0.001
Job strain								
Low			Referent				Referent	
Middle			1.61 (1.18–2.19)	0.002			1.65 (1.16–2.34)	0.004
High			1.82 (1.06–3.11)	0.031			1.75 (0.96–3.18)	0.074
Occupation support								
Low			Referent				Referent	
Middle			0.88 (0.51–1.52)	0.638			0.99 (0.57–1.73)	0.973
High			0.78 (0.44–1.39)	0.404			0.81 (0.45–1.48)	0.502
Burnout								
No			Referent				Referent	
Yes			1.89 (1.28–2.78)	0.001			1.87 (1.23–2.84)	0.004
Breadwinner								
No			Referent				Referent	
Yes			1.33 (1.10–1.60)	0.003			1.32 (1.09-−1.59)	0.004
Living condition								
Single			Referent				Referent	
With family or friend			1.13 (0.85–1.50)	0.414			1.17 (0.88–1.57)	0.284
Childcare or disabled family								
No			Referent				Referent	
Yes			1.23 (0.91–1.65)	0.178			1.24 (0.91–1.68)	0.172
Caregiver								
No			Referent				Referent	
Yes			1.54 (1.05–2.24)	0.026			1.46 (1.01–2.15)	0.041

*VDT, visual display terminal*.

*Model 1, adjusted for age, education, smoking, alcohol, exercise, and BMI*.

*Model 2, Model 1 + adjusted for department, seniority, working hours, shift work, VDT using at work, job strain, occupation support, and burnout*.

*Model 3, Model 1 + adjusted for breadwinner, living condition, child and disabled family, and caregiver*.

*Model 4, Model 2 + 3*.

*P-value was derived from multiple logistic regression analyses*.

### The Association Between Insomnia and Work/Family Characteristics by Using Multiple Logistic Regression Analysis According to sex and the Interaction Effect

Table 4 showed the results that revealed obvious differences between men and women in all relevant work characteristics and family responsibilities. Significantly higher ORs were noted in women with insomnia among those who had seniority more than 4 years (OR = 1.35, 95% CI: 1.05–1.73, *p* = 0.024; P _interactionforsex:_ 0.021), who worked more than 50 h/week (OR = 1.95, 95% CI: 1.24–3.06, *p* = 0.004; P _interactionforsex:_ 0.045), shift workers (OR = 1.32, 95% CI: 1.08–1.60, *p* = 0.013; P _interactionforsex_: 0.023), those with burnout syndrome (OR = 1.83, 95% CI: 1.20–2.81, *p* = 0.014; P _interactionforsex_: 0.032), those who were breadwinners (OR = 1.33, 95% CI: 1.09–1.62, *p* = 0.004; P _interactionforsex_: 0.033), and those who live with persons needing care and support (OR = 2.14, 95% CI: 1.15–8.98, *p* = 0.033; P _interactionforsex_: 0.024). Other factors, such as participants engaged in VDT used at work of more than 6 h/day, those with high job strain, those with low occupational support, and those who were living alone, were no statistical different for insomnia between different sex.

**Table 4:** The association between insomnia and work/family characteristics by using multiple logistic regression analysis according to sex and the interaction effect.

**Variables**	**Men**			**Women**				
	**OR**	**95% CI**	***p*-value**	**OR**	**95% CI**	***p*-value**	**P interaction for sex**
Department							0.307
Non-doctor/nurse	Referent					Referent	
Doctor/nurse		0.94	(0.44–1.98)	0.862	1.17	(0.69–1.49)	0.235
Seniority							0.021
<4 years	Referent					Referent	
≧4 years	1.15	(0.65–3.33)	0.351	1.35	(1.05–1.73)	0.024	
Working hours/week							0.045
<50 h/week	Referent					Referent	
≧ 50 h/week	1.10	(0.50–2.41)	0.812	1.95	(1.24–3.06)	0.004	
Shift work							0.023
No	Referent					Referent	
Yes	1.14	(0.54–2.38)	0.735	1.32	(1.08–1.60)	0.013	
Duration of VDT work							0.047
<6 h/day	Referent					Referent	
≧ 6 h/day	1.33	(1.10–1.62)	0.004	2.08	(1.00–4.33)	0.052	
Job strain (job demand/job control)							0.932
Low	Referent					Referent	
Middle	1.22	(0.13–3.12)	0.783	1.80	(0.27–2.39)	0.692	
High	1.19	(0.01–4.42)	0.305	1.35	(0.02–6.27)	0.484	
Occupation support							0.254
Low	Referent					Referent	
Middle	1.06	(0.22–5.00)	0.941	1.07	(0.46–4.45)	0.361	
High	0.76	(0.19–3.06)	0.703	0.88	(0.45–1.71)	0.705	
Burnout							0.032
No	Referent				Referent	
Yes	1.98	(1.08–3.14)	0.042	1.83	(1.20–2.81)	0.014	
Breadwinner							0.033
No	Referent					Referent	
Yes	1.04	(1.04–2.34)	0.025	1.33	(1.09–1.62)	0.004	
Living condition							0.583
Single	Referent					Referent	
With family or friend	1.14	(0.84–1.54)	0.411	1.38	(0.44–4.32)	0.583	
Childcare or disability family							0.707
No	Referent					Referent	
Yes	1.23	(0.89–1.69)	0.213	1.44	(0.49–4.25)	0.515	
Caregiver							0.024
No	Referent					Referent	
Yes	1.41	(0.94–2.10)	0.105	2.14	(1.15–8.98)	0.033	

*VDT, visual display terminal*.

*Adjusted for age, department, education, smoking, alcohol, exercise, BMI, seniority, working hours, shift work, VDT using at work, job strain, occupation support, burnout, breadwinner, living condition, child and disability family, and caregiver*.

*P-value was derived from multiple logistic regression analyses*.

## Discussion

We found that women had a higher prevalence of insomnia than men., In this study, seniority, working hours, shift works, VDT using at work, job strain, burnout, breadwinner status, living condition, and caregiver status were independently associated with insomnia. The gender difference in insomnia was strengthened after adjusting for work characteristics and family responsibilities. This result suggests that among HCWs in tertiary hospitals, gender differences in insomnia may be explained by gender differences in work characteristics and family responsibilities. The results of our stratified analyses showed that there were significant gender differences in insomnia only among HCWs under comparatively unfavorable circumstances. These results may indicate that work characteristics and family responsibilities have a far more significant influence on women with insomnia than men with insomnia among HCWs in tertiary hospitals.

The higher insomnia rate of HCWs working in tertiary hospitals, including up to 40% in India (28.6% were men and 41.0% were women) (7) and 48.5% in China (47.8% were men and 49.7% were women) (8). Another study of the United States of America (USA) front-line HCWs who had recently participated in the treatment of patients with coronavirus disease 2019 revealed that the high-risk groups for insomnia were women (707/963, 73.4%), individuals aged 30–49 years (692/963, 71.9%), and doctors (620/963, 64.4%) (6). Our study found that the proportion of insomnia was as high as 60.3% (52.7% were men and 61.7% were women), which was higher than the previous study results in China and India (7, 8) but less than those in the USA (6). The reasons for this difference were as follows 1. The assessment insomnia scales were different: we assessed insomnia using the Chinese Version of the Athens Insomnia Scale (CAIS) in our study. CAIS is based on the ICD-10 criteria and validated (32). The CAIS was a better screening tool than the India (Insomnia Severity Index) and China study (one insomnia question). 2. The age of the participants was different: the study group in India focused on 21–25-year-olds (7), and the mean age of our study showed 39.9 years in men and 38.24 years in women. The proportion of participants aged under 40 years in our study was less than that in the study in China (55.2% vs. 67.1%) (8) but similar in the study in the USA (30–49 years: 71.9%) (6). In other words, our participants and the USA front-line HCWs were slightly older than those studied in the other two tertiary hospitals.

In previous studies (6–8), it was consistently found that the proportion of women who had insomnia was higher than that of men, which was consistent with our findings. Furthermore, this was consistent with the results obtained in different countries, including the general population, or various jobs and assessments for measuring insomnia (6, 28, 36, 41–44). Our study among HCWs in tertiary hospitals found that women aged < 39 years had a higher prevalence of insomnia than other age and gender groups. This result was consistent with that of Yoshioka et al.'s study (Japanese women aged < 39 years) (36) and Liu et al.'s study of HCWs in Chinese tertiary hospitals (30–40 years old, regardless of gender) (8), but younger in the general population in Hong Kong and Korea (45–65 years and 40–49 years respectively) (42, 44). Therefore, the factors we explored, including work characteristics and family responsibilities, are essential for women HCWs. Consequently, it is necessary to develop appropriate intervention programs for HCWs in health care centers, especially in tertiary hospitals, to reduce the incidence of insomnia and improve working conditions.

Whether sociological factors can explain gender differences in insomnia symptoms (19, 20, 36), a national study in Taiwan found that women scored 1.25 points higher than men on the insomnia inventory (*p* < 0.001), after controlling for family factors, women's sleep quality was still significantly worse than men's. Still, after controlling for social roles, the gender differences in insomnia decreased slightly (19). In another study in Hong Kong and Greater Stockholm area, they were found that after adjusting for various demographic and lifestyle factors, the gender difference in insomnia was significant (44, 45). Although these studies have limited work characteristics and family responsibilities that can be evaluated, there is still relatively consistent support for the impact of gender differences in insomnia.

Sekine et al. (28), Yoshioka et al. (36), and Doi et al. (41) analyzed employees working in the local government and at a telecom company in Japan and found that when they adjusted for work characteristics and family responsibilities, gender differences were weakened and no longer significant. However, the participants in these studies are in similar workplaces, and their social conditions make them a relatively homogeneous population. Our research evaluated and investigated the gender differences in insomnia among HCWs in a tertiary hospital, which was a different kind of job from previous studies. After adjusting for work characteristics and family responsibilities, gender advantages remained significant.

Family socioeconomic status also influences gender differences in insomnia. Martikainen et al. indicated that analyses of the socioeconomic gradient in women's health in Japan might better predict insomnia (46). Yoshioka et al. suggested that the health of female workers is influenced more by household income (36). In our study, women who were breadwinners had higher rates of insomnia than women who were not. According to these results, stratifying the subgroup analysis by gender still has an impact on gender differences in our study.

Shift work causes sleep disorders (19, 28, 47). Our results showed that women with shift works had a higher association for insomnia than those without shift works. Another finding was that women living alone had a higher association of insomnia than women living with family. These results are consistent with those of previous studies (5, 8, 48). A systematic review by Brito et al. reported that the prevalence of insomnia in shift workers ranged from 12.8 to 76.4%, higher than the estimated rate for the general population.

Moreover, a higher prevalence was observed in women and people living alone (48). HCWs usually need to provide 24/7 service; thus, working longer shifts (including night shifts) and longer hours. This mode of work causes sleep deprivation (8, 49). In addition, night shifts cause internal circadian rhythm disorders and confusion in the work-rest schedule, leading to aggravation of insomnia.

Cross-sectional studies in China and the US found that long working hours are a predictor of insomnia (8, 50). As working hours increase, sleep duration decreases, and sleep quality declines (8, 50). Hale et al. proved that people working more than 50 h/week are more likely to be short sleepers (51), while Liu et al. indicated that HCWs in tertiary hospitals who work more than 55 h/week have an increased risk of insomnia (8). Our study found the same result, and women were at a higher risk than men. Many countries have explicit rules that limit the working hours of HCWs. For example, the European Union in 2009 and Taiwan in 2012 stipulated a maximum of 48 h of work per week and defined rest periods (52, 53).

A study of the Chinese medical staff found that 46 cases of karoshi or death by overwork occurred from 2013 to 2015, most of which were women aged 30–39 years (54). The causes included continuous exposure of HCWs to the risk of insomnia and lack of sleep, irregular exercise, and unhealthy eating habits, which could increase the risk of chronic diseases. A previous Chinese study also pointed out that burnout is a significant signal of karoshi of physicians in China (55). Our study found that burnout increases the risk of insomnia. Additionally, it had a more powerful influence on women than on men. Previous studies have shown a significant association between VDT used at work and sleep disorders (56, 57). However, our study found a significant association and higher association of insomnia among women with VDT used at work of more than 6 h/day than among men with the exact duration of VDT used at work.

As defined by the demand-control-support model, the effect of being under high level of stress is widely used as a risk factor for sleep disturbance in theoretical occupational stress models (28, 58, 59). In our study, women had higher job strain and lower occupational support than men. The results of our research are convincing because they are like the outcomes of previous research suggesting high risks of insomnia owing to high demands, low control, and low social support, especially in nurses (60–62).

An analysis of 9,851 Hong Kong Chinese individuals (46.4% males; 53.6% females) revealed that the risk of insomnia in women was 1.6 times that in men (44). Multivariate analysis showed that family financial pressure was related to an increased risk of insomnia in both sexes. Our stratified research and analysis showed similar results. Compared with men HCWs, women HCWs had a higher risk of insomnia caused by breadwinner status, and caregiver roles.

Our study had several strengths. First, we used highly reliable scales such as CAIS-8 (32, 33), MBI-HSS (34), DCS model (29, 37, 38), and questionnaires created in 2016 from the Institute of Labor, Occupational Safety and Health, Ministry of Labor in Taiwan (ILOSH105–M309) (31), all of which have a high degree of credibility. Reliability and validity were confirmed. Second, previous studies mentioned that anxiety and depression affected sleep quality and induced insomnia (22, 63, 64). This study excluded participants with anxiety and depression, with or without treatment, to avoid the impact on our results. Third, in this study, 78.5% of participants were aged <50 years (before menopause); thus, insomnia caused by hormone differences could be excluded. Fourth, Li et al. reported that smoking habits were confounded with insomnia in women (44). Smoking and alcohol habits were adjusted in this study. Finally, this study explored the prevalence of insomnia among different genders among HCWs in tertiary hospitals. Itassessed the roles of work characteristics and family responsibilities, which are currently less explored.

Several limitations should be considered when interpreting our results. First, there was a significant difference in the sample size between men (317, 11.3%) and women (2,494, 88.7%), mainly because of female nursing staff. Although the chi-squared test showed significant differences between the proportions of men and women for many variables, there were some restrictions for men when stratifying the analysis by age and gender because of the small sample size. Second, all participants were from different branches of the two tertiary hospitals. There is indeed an intraclass correlation. However, in the multiple regression analysis in Tables 3, 4 of this study, there is no statistical difference between the classified doctor/nurse and non-doctor/nurse groups, so the intraclass correlation has little effect. That is also the Neyman bias in this study. Therefore, the study results may not be generalizable to other HCWs. In the future, data should be collected from multiple hospitals and regions, and studies should include more male participants. Third, because this was a cross-sectional study, the causal nature of the association between insomnia and work characteristics and family responsibilities could not be determined. Fourth, the variables that could be analyzed were limited. Relevant information about work, family, and tea/coffee consumption were obtained through self-answered questionnaires, which were not reliable and might have caused reporting bias. Missing confounding factors, such as light exposure, napping during shifts, previous diagnosis of sleep disorders, and medication use, could not obtain from this study. Finally, this study was a voluntary response sample, and there was a problem of self-selection bias.

## Conclusion

Our study showed that women HCWs in tertiary hospitals had higher insomnia than men HCWs. The gender difference was strengthened and still significant after adjusting for work characteristics and family responsibilities. These results suggest that gender differences in insomnia are explained primarily by gender differences in work characteristics and family responsibilities. Women had a higher risk of insomnia than men, especially among HCWs under comparatively unfavorable circumstances. To clarify the roles of work characteristics and family responsibilities on gender differences in insomnia, further investigation is needed using a more detailed sociological approach. Women HCWs in tertiary hospitals must consider their work characteristics and family responsibilities to avoid physical and mental health effects of insomnia and its impact on work efficiency and patient care quality.

## Data Availability Statement

The original contributions presented in the study are included in the article/supplementary material, further inquiries can be directed to the corresponding author.

## Ethics Statement

The studies involving human participants were reviewed and approved by the Research Ethics Committee on Human Beings at MacKay Memorial Hospital (Research Project Number 18MMHISO150). The patients/participants provided their written informed consent to participate in this study.

## Author Contributions

M-TT participated in the design and conception of the study and its coordination and data acquisition, and also performed statistical analysis and drafted the manuscript. The author reviewed the manuscript and read and agreed to the published version of the manuscript.

## Conflict of Interest

The author declares that the research was conducted in the absence of any commercial or financial relationships that could be construed as a potential conflict of interest.

## Publisher's Note

All claims expressed in this article are solely those of the authors and do not necessarily represent those of their affiliated organizations, or those of the publisher, the editors and the reviewers. Any product that may be evaluated in this article, or claim that may be made by its manufacturer, is not guaranteed or endorsed by the publisher.
